# Latent profile analysis of kinesiophobia and influencing factors among middle-aged stroke patients during the recovery period

**DOI:** 10.3389/fpsyg.2026.1975436

**Published:** 2026-10-26

**Authors:** Ting Liu, Xiaosui Yang, Zhixia Zhang, Yaqiong Hu

**Affiliations:** 1 Department of Otolaryngology, Tianyou Hospital Affiliated to Wuhan University of Science and Technology, Wuhan, Hubei, China; 2 Department of Scientific Research, Tianyou Hospital Affiliated to Wuhan University of Science and Technology, Wuhan, Hubei, China; 3 Department of Spine and Bone Oncology, Zhongnan Hospital of Wuhan University, Wuhan, Hubei, China

**Keywords:** influencing factors, kinesiophobia, latent profile analysis (LAT), middle-aged, stroke

## Abstract

**Objective:**

To explore latent kinesiophobia profiles in middle-aged stroke patients during the recovery phase and to identify factors associated with profile membership.

**Methods:**

This cross-sectional study employed convenience sampling to recruit middle-aged stroke patients in the recovery phase from six tertiary hospitals in Wuhan between May 15 and September 15, 2025. Patients completed a general information questionnaire, the Tampa Scale of Kinesiophobia (TSK), the Stroke Symptom Experience Scale (Stroke-SES), and the Confidence after Stroke Measure (CaSM). Latent profile analysis (LPA) was used to identify latent kinesiophobia profiles. Multinomial logistic regression analysis was performed to examine factors associated with different profiles.

**Results:**

A total of 364 questionnaires were returned, of which 353 were valid (96.98%). LPA delineated three latent kinesiophobia profiles among middle-aged stroke patients: the self-regulation profile (15.6%), pain-dominated profile (64.9%), and trauma-sensitive profile (19.5%). Multinomial logistic regression identified education level, family history of stroke, number of episodes, symptom burden, and rehabilitation confidence as significant differentiating factors across these profiles (all *p* < 0.05).

**Conclusion:**

Kinesiophobia levels vary considerably across middle-aged stroke patients in the recovery phase. Healthcare providers should pay attention to the distinct kinesiophobia patterns and demographic characteristics of different patients and develop targeted interventions to reduce kinesiophobia and promote active participation in exercise rehabilitation.

## 1 Introduction

As the second leading cause of death globally and the leading cause of disability in Chinese adults (Hilkens et al., 2024), stroke affected 101 million people worldwide in 2019, representing 11.6% of all deaths (Ma et al., 2021). In China, approximately 2.5 million new cases are reported each year, resulting in over 1.5 million deaths and a 75% disability rate among survivors (Tu and Wang, 2023). Notably, the incidence of stroke among middle-aged adults in China has exhibited a consistent upward trajectory (Lu et al., 2021), with this age group accounting for 71.80% of individuals at high risk for stroke (Report on Stroke Prevention and Treatment in China Writing Group, 2023). As the primary breadwinners and key drivers of social development, these individuals not only face considerable financial strain after stroke but also represent a substantial loss of workforce productivity. This dual burden underscores the urgency of addressing the clinical gaps in this patient group.

Exercise rehabilitation has been shown to improve neurological and motor functions, reduce readmission and mortality rates, and enhance prognosis and quality of life in post-stroke patients (Lee et al., 2022). Unfortunately, many stroke survivors do not adhere to exercise regimens, with compliance to medically supervised exercise rehabilitation falling below 35% (Choi et al., 2022). In particular, a previous study demonstrated that early application of rehabilitation exercise after stroke is effective (Liu et al., 2022). The recovery phase following a stroke is a critical period for restoring and rebuilding impaired neurological functions (Zhang et al., 2023). However, while acute-phase care prioritizes physical and occupational therapy, exercise-based rehabilitation is often overlooked in the early recovery period. Sütçü Uçmak and Kılınç (2024) identified kinesiophobia as a key psychological factor underlying patient inactivity, which in turn poses a major obstacle to early stroke rehabilitation.

Kinesiophobia, first introduced by Kori, refers to an exaggerated and irrational avoidance of physical activity, rooted in the perception that exercise may cause pain or re-injury (Kori, 1990). According to previous studies, approximately 80% of stroke patients experience kinesiophobia (Wasiuk-Zowada et al., 2021; Bąk et al., 2022), and those with more severe symptoms tend to be less physically active (Sütçü Uçmak and Kılınç, 2024). As an independent factor, kinesiophobia directly affects adherence to exercise rehabilitation among stroke patients, leading to declines in aerobic capacity and muscle strength, as well as an increased risk of recurrent stroke and mortality (Sütçü Uçmak and Kılınç, 2024). However, previous investigations into stroke-related kinesiophobia have largely centered on older adults, with insufficient attention given to middle-aged patients. More critically, prior work has predominantly relied on aggregate scale scores to gauge overall kinesiophobia, thereby overlooking inter-individual differences within this population.

Latent profile analysis (LPA) applies probabilistic models to classify individuals with shared symptom characteristics into distinct latent profiles, thus better reflecting inter-individual heterogeneity (Spurk et al., 2020). In a study of 252 patients with coronary heart disease, Wang et al. (2023) identified three kinesiophobia profiles via LPA; the moderate profile accounted for 62.7%, and older age was linked to a higher probability of high-kinesiophobia profile membership. Currently, LPA is widely applied in psychology, medicine, and other fields. However, research on the classification of kinesiophobia specifically in stroke patients remains scarce. Consequently, this study used LPA to classify kinesiophobia in middle-aged stroke patients and examine profile-specific influencing factors, with the goal of guiding tailored intervention strategies for distinct patient profiles.

## 2 Materials and methods

### 2.1 Participants

Using the convenience sampling, we selected two tertiary general hospitals from each of the three major districts of Wuhan (Wuchang, Hankou, and Hanyang), Hubei Province, between May 15 and September 15, 2025. The study enrolled middle-aged stroke patients in the recovery phase from the Departments of Neurology and Rehabilitation Medicine at the six participating hospitals. Inclusion criteria: (1) stroke survivors meeting the diagnostic criteria established by the Chinese Medical Committee(Zhang et al., 2023); (2) age 45–59 years, per the WHO definition of middle age; (3) in the recovery phase of stroke (disease duration of 15 days to 6 months), according to the Chinese Stroke Association guidelines(Zhang et al., 2023); (4) Barthel Index score > 41, ensuring sufficient functional independence to participate in exercise-based rehabilitation(Quinn et al., 2011); (5) conscious and able to communicate effectively; and (6) providing informed consent and volunteering to participate. Exclusion criteria: (1) organic disorders of major organs (e.g., heart, liver, kidneys); (2) comorbid mental disorders; and (3) comorbidity with severe skeletal musculoskeletal pathology or other disorders that affect exercise function. Functional independence was assessed by trained clinicians using the Barthel Index as part of routine clinical care during the recovery phase. This assessment was used solely for eligibility screening (Barthel Index > 41); exact Barthel Index scores were not systematically recorded as study variables.

This cross-sectional study estimated the minimum sample size using the formula: n = μα/22σ2/δ2 (Xie et al., 2025), setting α = 0.05 (μα/2 = 1.96), δ = 1.5, and σ = 10.2. After allowing for a 20% attrition rate, the required sample size was determined as 213. In addition, to further justify the sample size for LPA, we considered methodological recommendations for mixture modeling. Previous studies suggested that a minimum of 300 cases is generally recommended for LPA, and that with adequate profile separation, a sample of around 500 provides sufficient power to detect the correct number of profiles (Nylund-Gibson et al., 2023). In this study, a total of 353 participants were included, which provided adequate power for the LPA. Furthermore, the smallest profile in our three-profile solution comprised 15.6% of the total sample (*n* = 55), well above the commonly recommended 5% threshold (Nylund-Gibson et al., 2023), and entropy was 0.916, indicating adequate class separation.

### 2.2 Measures

#### 2.2.1 General information questionnaire

This questionnaire was designed by the researcher and collected data on gender, age, education level, marital status, monthly income, comorbidities, and family history of stroke.

#### 2.2.2 Tampa scale of Kinesiophobia (TSK)

This scale was originally designed by Vlaeyen et al. (1995) and later translated and culturally adapted for Chinese populations by Hu (2012). It contains 17 items using a 4-point Likert scale; total scores range from 17 to 68, with higher scores indicating more severe kinesiophobia and a cutoff of ≥37 defining high levels. Its Chinese version has been validated in chronic illnesses (Hu, 2012), with Cronbach’s alpha values of 0.778 and 0.960 in prior and our studies, respectively.

#### 2.2.3 Stroke symptom experience scale (stroke-SES)

This scale was developed by Shi et al. (2019) in 2019 and is a 19-item scale widely used in China to evaluate stroke-related symptoms. Each item is rated across three dimensions: frequency (scores 1–4), severity (scores 1–4), and distress (scores 0–4). The symptom burden score for each item is calculated as the mean of its three dimension scores. The overall symptom burden score is computed by summing the item-level burden scores across all 19 items, with a possible range of 12.67 to 76; higher scores indicate greater symptom burden. The Cronbach’s alpha values were 0.810 for the original scale and 0.918 in this study.

#### 2.2.4 Confidence after stroke measure (CaSM)

This scale was originally developed by Horne et al. (2017) in 2017 and was localized and revised by Hu et al. (2018) in 2018. This 27-item scale comprises three domains (self-confidence, positive attitude, and social confidence), using a 4-point Likert format. Of the 27 items, 17 are negatively worded (Items 1–4, 6–9, 18–25, and 27), scored 0 = completely agree to 3 = completely disagree, and 10 are positively worded (Items 5, 10–17, and 26), reverse-scored so that 3 = completely agree and 0 = completely disagree. After reverse scoring, all items are summed to yield a total score ranging from 0 to 81, with higher scores reflecting stronger confidence. Its Chinese version has been validated in chronic disease cohorts (Song, 2025), with Cronbach’s alpha values of 0.974 and 0.910 in prior and our studies, respectively.

### 2.3 Data collection

This study used paper-based, face-to-face questionnaires. Prior to data collection, we obtained institutional approval and selected one principal investigator and one data collector from each hospital to undergo standardized training. Trained investigators briefed eligible patients on the study aims and key considerations prior to obtaining informed consent, after which participants completed the questionnaires with anonymity ensured. Investigators clarified queries during administration, and for patients with visual or other limitations, items were read aloud and answers recorded by the investigator. To limit subjective bias in such cases, only investigators free of conflicts of interest were assigned to this role. All completed forms were inspected on-site for incompleteness, inaccuracies, or multiple selections. Questionnaires were deemed invalid and excluded from the final analysis if all responses were identical or if more than 10% of the items were left blank. Throughout the study, the heads of each participating hospital regularly reported common and site-specific issues encountered, which were promptly resolved.

### 2.4 Statistical analysis

Latent profile analysis (LPA) was conducted using Mplus 8.3 (Muthén & Muthén, Los Angeles, CA, USA), with one- to five-profile models fitted sequentially. Model fit was assessed by Akaike Information Criterion (AIC), Bayesian Information Criterion (BIC), adjusted BIC (aBIC), entropy, and the Lo–Mendell–Rubin adjusted likelihood ratio test (LMRT) and bootstrap likelihood ratio test (BLRT). Lower AIC, BIC, and aBIC values reflect better fit; entropy values approaching 1 indicate higher classification precision. The LMRT and BLRT compare a k-profile model against a k-1-profile model, with a significant result (*p* < 0.05) favoring the k-profile solution. Descriptive and comparative analyses were performed using SPSS 26.0 (IBM Corp., Armonk, NY, USA). Categorical variables were reported as frequencies (percentages), and continuous variables as mean ± SD after normality checking; otherwise, non-parametric tests were used. Intergroup differences were assessed using chi-square and one-way analysis of variance (ANOVA), and multinomial logistic regression was fitted to explore correlates of kinesiophobia profiles. To control for potential confounders, all variables that were statistically significant in univariate analyses were entered simultaneously into the multinomial logistic regression model. Given the exploratory design, we prioritized univariately significant variables to maintain model parsimony. This approach allowed each variable to be adjusted for the effects of all other variables in the model. A two-sided *p* < 0.05 was considered statistically significant.

## 3 Results

### 3.1 Descriptive statistics

A total of 353 valid responses were retrieved from 364 distributed questionnaires (response rate: 96.98%). The participants consisted predominantly of male patients (59.2%), with a mean age of 49.33 ± 3.94 years. In terms of sociodemographic characteristics, the most common profiles were education ≤ junior high school (38.5%), married (89.2%), self-employed (42.2%), partner as primary caregiver (50.1%), and monthly income of 3,000–5,000 RMB (34.3%). Clinically, over 62.3% of patients had ischemic stroke, 67.7% reported a family history of stroke, nearly half (45.9%) had no complications, and 31.4% had experienced four or more stroke episodes. In addition, the mean total scores were 44.89 ± 7.87 for the TSK scale, 30.39 ± 13.27 for the Stroke-SES scale, and 62.04 ± 11.15 for the CaSM scale. The CaSM subscale scores were as follows: self-confidence, 21.08 ± 4.72; positive attitude, 19.39 ± 5.28; and social confidence, 21.57 ± 5.05.

### 3.2 Potential profile analysis results

Using TSK item scores as indicators, we performed LPA to identify kinesiophobia profiles in middle-aged stroke patients during the recovery phase. Models with one to five profiles were fitted sequentially, with fit indices summarized in Table 1. AIC, BIC, and aBIC values exhibited consistent reductions across models, and entropy remained above 0.8 for all profiles. However, given the non-significant LMRT for the 4- and 5-profile models (*p* > 0.05), the 3-profile solution was retained as the best balance of statistical fit and clinical utility.

**Table 1:** Fit indices for latent profile models of kinesiophobia in middle-aged stroke patients (*n* = 353).

Model	AIC	BIC	aBIC	Entropy	LMRT	BLRT	Class probability
1	15851.865	15983.325	15875.463	—	—	—	—
2	15190.584	15391.640	15226.675	0.905	<0.001	<0.001	0.979/0.963
3	14803.162	15073.815	14851.747	0.916	<0.001	<0.001	0.936/0.973/0.947
4	14656.972	14997.222	14718.05	0.877	0.356	<0.001	0.916/0.899/0.935/0.991
5	14580.332	14990.178	14653.903	0.874	0.175	<0.001	0.967/0.874/0.928/0.987/0.889

AIC, Akaike Information Criterion; BIC, Bayesian Information Criterion; aBIC, adjusted BIC; LMRT, Lo–Mendell-Ruben adjusted likelihood ratio test; BLRT, bootstrap likelihood ratio test.

### 3.3 Profile naming and characteristics

Based on TSK item scores, LPA identified three distinct kinesiophobia profiles (Figure 1). Profile 1 was labelled the “self-regulation profile” (15.6%), with significantly lower total scores and lower scores across all dimensions compared with the other two profiles, particularly on items reflecting avoidance of movement and fear of re-injury (e.g., items 9 and 10). Profile 2, termed the “pain-dominated profile” (64.9%), exhibited elevated scores on items related to pain-related activity avoidance (e.g., items 7 and 8). Profile 3 was designated the “trauma-sensitive profile” (19.5%), with overall scores significantly higher than those of the other two profiles. Notably, however, this group scored significantly lower on item 5 (“This unexpected health issue could jeopardize my future health.”).

**Figure 1:**
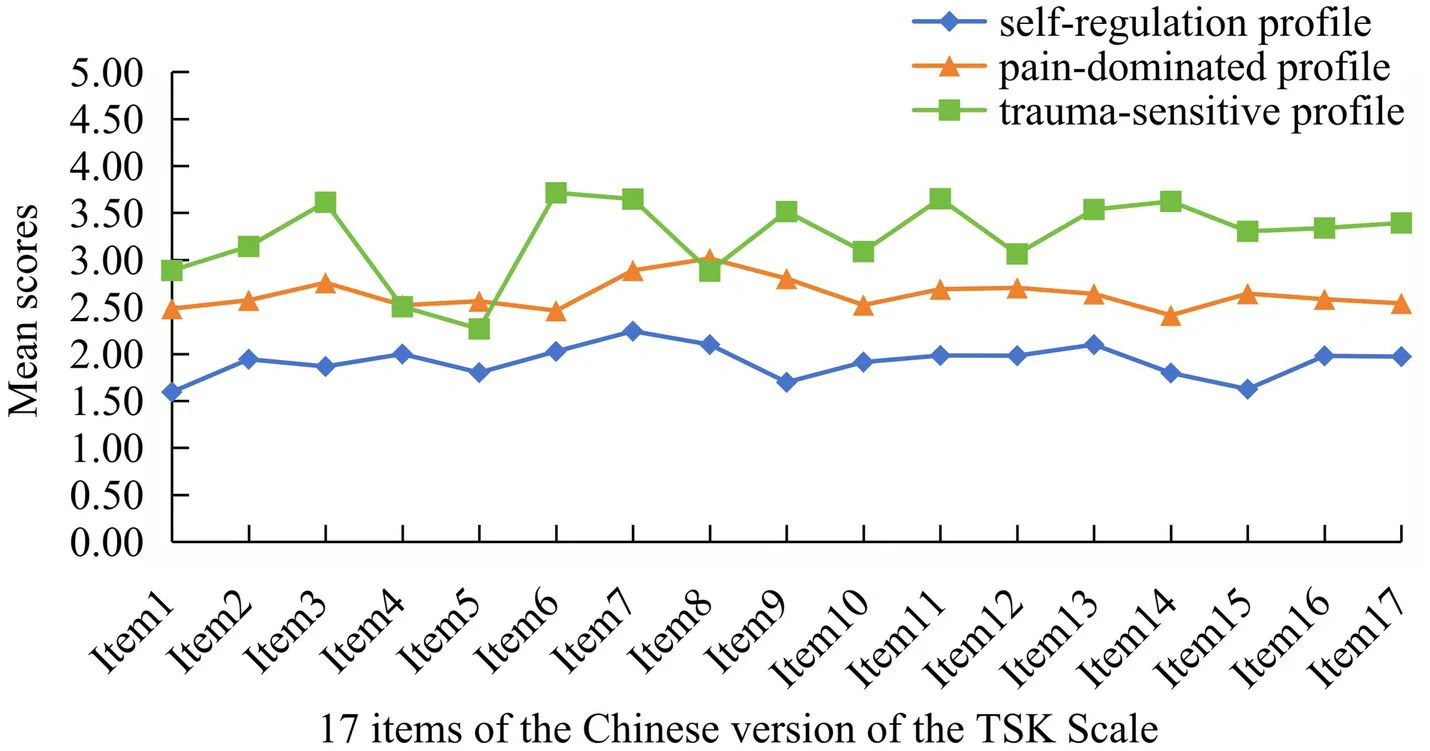
Item score profiles of the three latent kinesiophobia profiles among middle-aged stroke patients.

The three profiles were named based on their item-level response patterns, and do not represent clinical categories defined by the TSK cutoff of ≥37. The self-regulation profile was characterized by relatively low scores across all 17 items. The pain-dominated profile showed selectively elevated scores on items concerning pain-related avoidance. The trauma-sensitive profile displayed high overall scores but a distinctively lower score on item 5. In this study, the mean total TSK scores for the three profiles were as follows: self-regulation profile, 32.55 ± 4.20; pain-dominated profile, 44.76 ± 3.63; and trauma-sensitive profile, 55.16 ± 5.69. The proportions of participants with TSK ≥ 37 within each profile were 21.8% (*n* = 12), 99.1% (*n* = 227), and 100.0% (*n* = 69), respectively.

### 3.4 Univariate analysis of factors associated with kinesiophobia profiles among middle-aged stroke patients

Univariate analysis showed significant differences in education level, monthly income, family history of stroke, number of episodes, symptom burden, and rehabilitation confidence across the three kinesiophobia profiles (*p* < 0.05) (Table 2).

**Table 2:** Demographic characteristics across kinesiophobia latent profiles in middle-aged stroke patients (*n* = 353).

Variables	Self-regulation profile (*n* = 55)	Pain-dominated profile (*n* = 229)	Trauma-sensitive profile (*n* = 69)	χ2/F	*p*
Age	49.60 ± 3.81	48.98 ± 3.68	50.25 ± 4.70	2.918	0.055
Gender, *n* (%)				0.841	0.657
Male	33 (60.0)	132 (57.6)	44 (63.8)		
Female	22 (40.0)	97 (42.4)	25 (36.2)		
Education level, *n* (%)				97.218	<0.001
Junior high school or below	0 (0.0)	94 (41.1)	42 (60.9)		
High school	14 (25.5)	80 (34.9)	27 (39.1)		
Undergraduate or above	41 (74.5)	55 (24.0)	0 (0.0)		
Marital status, *n* (%)				4.634	0.327
Unmarried	2 (3.6)	4 (1.7)	0 (0.0)		
Married	46 (83.6)	208 (90.8)	61 (88.4)		
Divorced	7 (12.8)	17 (7.5)	8 (11.6)		
Occupation type, *n* (%)				5.549	0.476
Farmer	8 (14.5)	19 (8.3)	4 (5.8)		
Office worker	19 (34.5)	93 (40.6)	26 (37.7)		
Self-employed	20 (36.5)	97 (42.4)	32 (46.4)		
Unemployed	8 (14.5)	20 (8.7)	7 (10.1)		
Caregiver, *n* (%)				11.241	0.081
Living alone	2 (3.6)	8 (3.5)	2 (2.9)		
Children	11 (20.0)	44 (19.3)	22 (31.9)		
Partner	35 (63.6)	113 (49.3)	29 (42.0)		
Caregivers or others	7 (12.8)	64 (27.9)	16 (23.2)		
Monthly income (RMB), *n* (%)				101.841	<0.001
<3,000	0 (0.0)	92 (40.2)	25 (36.2)		
3,000–5,000	12 (21.8)	65 (28.4)	44 (63.8)		
>5,000	43 (78.2)	72 (31.4)	0 (0.0)		
Type of stroke, *n* (%)				3.775	0.151
Ischemic	36 (65.5)	148 (64.6)	36 (52.2)		
Hemorrhage	19 (34.5)	81 (35.4)	33 (47.8)		
Family history of stroke, *n* (%)				53.295	<0.001
Yes	14 (25.5)	174 (76.0)	51 (73.9)		
No	41 (74.5)	55 (24.0)	18 (26.1)		
Number of comorbidities, *n* (%)				11.141	0.084
0	29 (52.7)	111 (48.5)	22 (31.9)		
1	18 (32.8)	85 (37.1)	28 (40.6)		
2	6 (10.9)	29 (12.7)	15 (21.7)		
≥3	2 (3.6)	4 (1.7)	4 (5.8)		
Number of episodes, *n* (%)				77.869	<0.001
1	26 (47.3)	33 (14.5)	0 (0.0)		
2	16 (29.1)	53 (23.1)	6 (8.7)		
3	8 (14.5)	63 (27.5)	37 (53.6)		
≥4	5 (9.1)	80 (34.9)	26 (37.7)		
Stroke-SES	14.90 ± 8.23	29.72 ± 6.42	44.93 ± 17.58	143.254	<0.001
CaSM	67.25 ± 6.04	65.74 ± 6.82	45.61 ± 10.78	198.557	<0.001
TSK	32.55 ± 4.20	44.76 ± 3.63	55.16 ± 5.69	—	—

Stroke-SES, scores of the Stroke Symptom Experience Scale; CaSM, scores of the Confidence after Stroke Measure; TSK, scores of the Tampa Scale of Kinesiophobia.

### 3.5 Multicollinearity assessment

To evaluate the suitability of selected explanatory and control variables for regression analysis, variance inflation factors (VIF) were computed to quantify multicollinearity. In this study, the VIF values ranged from 1.138 to 1.433 (Table 3), meeting the criterion that VIF values should fall between 0 and 5, thereby confirming that there is no significant multicollinearity among the independent variables.

**Table 3:** VIF analysis.

Variables	VIF	1/VIF
Education level	1.433	0.698
Monthly income (RMB)	1.138	0.878
Family history of stroke	1.211	0.826
Number of episodes	1.219	0.820
Stroke-SES	1.359	0.736
CaSM	1.216	0.823

Stroke-SES, scores of the Stroke Symptom Experience Scale; CaSM, scores of the Confidence after Stroke Measure.

### 3.6 Multinomial logistic regression analysis of kinesiophobia among middle-aged stroke patients

Using the self-regulation profile as a reference, multinomial logistic regression was performed with all significant univariate predictors as independent variables. The coding scheme for variables was as follows: (1) Education level: Junior high school or below = 1, High school = 2, Undergraduate or above = 3; (2) Monthly income: <3,000 = 1, 3,000–5,000 = 2, >5,000 = 3; (3) Family history of stroke: Yes = 1, No = 2; (4) Number of episodes: 1 = 1, 2 = 2, 3 = 3, ≥ 4 = 4. Symptom burden (Stroke-SES total score) and rehabilitation confidence (CaSM total score) were treated as continuous variables and entered directly into the model. In SPSS, the highest coded value was set as the reference category by default. The results showed that symptom burden, rehabilitation confidence, education level, family history of stroke, and number of episodes were significant discriminators across the three profiles (*p* < 0.05) (Table 4).

**Table 4:** Multinomial logistic regression results for kinesiophobia profiles among middle-aged stroke patients (*n* = 353).

Variables	β	SE	Wald χ^2^	*p*	OR	95% CI
Self-regulation profile vs. Pain-dominated profile						
Intercept	−4.470	4.304	1.079	0.299	—	—
Stroke-SES	0.289	0.057	26.088	<0.001	1.336	1.195 ~ 1.493
Education = High school	2.626	0.840	9.777	0.002	13.823	2.665 ~ 71.701
Family history of stroke = Yes	2.165	0.803	7.269	0.007	8.717	1.806 ~ 42.070
Number of episodes = 1	−2.284	1.285	4.829	0.028	0.059	0.050 ~ 0.737
Self-regulation profile vs. Trauma-sensitive profile						
Intercept	−35.361	4.680	57.092	<0.001	—	—
Stroke-SES	0.372	0.061	37.025	<0.001	1.451	1.287 ~ 1.635
CaSM	−0.255	0.071	12.778	<0.001	0.775	0.674 ~ 0.891
Number of episodes = 1	−3.230	1.609	4.031	0.045	0.041	0.002 ~ 0.926

Stroke-SES, scores of the Stroke Symptom Experience Scale; CaSM, scores of the Confidence after Stroke Measure.

Model fit was assessed using several indicators. The likelihood ratio test was statistically significant (χ^2^ = 463.908, df = 16, *p* < 0.001), indicating that the model with predictors fit significantly better than the intercept-only model. The pseudo R^2^ values were as follows: Cox and Snell = 0.731, Nagelkerke = 0.880, McFadden = 0.739. The classification table showed that 91.5% of cases were correctly classified. The Pearson chi-square test for goodness-of-fit was not significant (χ^2^ = 406.261, *p* > 0.05), indicating adequate model fit.

## 4 Discussion

### 4.1 Kinesiophobia levels in middle-aged stroke patients

The mean TSK score in this study was 44.89 ± 7.87, indicating a relatively high level of kinesiophobia. Overall, 84.4% of patients fell into the pain-dominated or trauma-sensitive profiles, consistent with Gu et al. (2026), though the pain-dominated profile comprised a substantially larger proportion. This discrepancy may be due to our specific focus on middle-aged patients. Relative to elderly patients, middle-aged individuals typically confront greater family and societal demands (Frei et al., 2025), which may foster greater exercise adherence to facilitate early reintegration into daily life. Wasiuk-Zowada et al. (2021) found that ~70% of stroke patients exhibit kinesiophobia, with irrational fear and activity avoidance stemming from excessive worry about exercise-induced harm. Such fear-avoidance behaviors can jeopardize rehabilitation adherence and impede recovery.

Importantly, “self-regulation profile,” “pain-dominated profile,” and “trauma-sensitive profile” are relative LPA-derived profiles based on item-level patterns in our study, rather than categories defined by the TSK cutoff of ≥37. Despite a mean TSK score above 37, only 19.5% were in the trauma-sensitive profile; TSK ≥ 37 proportions across profiles were 21.8%, 99.1%, and 100.0%. Thus, 84.4% reflects the combined pain-dominated and trauma-sensitive LPA profiles, not clinically defined high kinesiophobia. These profiles remain relative and data-driven unless validated against clinical cutoffs. These findings highlight the heterogeneity of kinesiophobia in this population and suggest that profile-based assessment may be worth exploring.

### 4.2 Heterogeneity of kinesiophobia in middle-aged stroke patients

The LPA identified three distinct kinesiophobia profiles among middle-aged stroke patients during the recovery phase, suggesting marked heterogeneity in fear levels. (1) The “self-regulation profile” (15.6%) scored lower on items assessing avoidance behavior and fear of re-injury than the other two profiles (e.g., items 9 and 10), which may suggest adequate awareness of exercise-related risks and sufficient self-regulatory capacity. (2) The “pain-dominated profile” (64.9%) exhibited higher scores on items related to pain-related activity avoidance (e.g., items 7 and 8); these patients were more likely to resist exercise due to fear of re-injury or anticipated pain. (3) The “trauma-sensitive profile” (19.5%) showed generally high item scores but scored notably lower on item 5 (“This unexpected health issue could jeopardize my future health”), suggesting that their heightened fear may be predominantly anchored in the traumatic stroke event itself, rather than in long-term health concerns. This pattern may be associated with further sedentary behavior and reduced engagement in rehabilitation. Therefore, timely assessment and profile-specific interventions may help improve rehabilitation engagement and outcomes in this group, although their effectiveness requires further investigation.

### 4.3 Influencing factors of kinesiophobia profiles in middle-aged stroke patients

This study found that, compared with junior high school or below, high school education was associated with a greater likelihood of classification into the “pain-dominated profile.” This finding appears inconsistent with a prior study (Miao et al., 2025), which reported that lower educational attainment is associated with greater fear of movement. Several factors may explain this discrepancy. Our sample was restricted to middle-aged stroke patients in the recovery phase, a group that may face unique occupational and family pressures. In addition, patients with a high school education might be more attentive to bodily sensations or have different expectations regarding recovery, which might be associated with greater pain-related avoidance without reaching the levels observed in the trauma-sensitive profile. Contrary to our expectation, higher educational attainment did not appear to facilitate a more objective appraisal of rehabilitation in this sample. Therefore, clinical strategies may need to account for educational differences and could consider providing tailored, stepwise health education and skills training to all patients, with particular attention to how those with a high school education interpret pain and movement-related sensations, with the aim of enhancing disease-related knowledge and health literacy.

Our results showed that patients with a family history of stroke were more likely to be classified into the “pain-dominated profile,” whereas those without such a family history exhibited lower levels of kinesiophobia, a finding inconsistent with Zhou et al. (2026). A previous study identified family history of stroke as a key factor associated with post-stroke exercise maintenance, as a parental or sibling history of stroke may be associated with anxiety and depression in patients, which in turn may foster irrational fear toward exercise rehabilitation (Aycock et al., 2015). Middle-aged patients, who bear heavier family responsibilities, are particularly susceptible to negative illness perceptions within their families and the financial burden associated with hereditary risks. These factors may be associated with excessive caution toward rehabilitation therapy, which may in turn be associated with greater fear of exercise. Therefore, active involvement of key family members in health education may be beneficial to correct misconceptions and negative attitudes toward exercise rehabilitation, potentially enhancing patients’ exercise adherence and rehabilitation confidence, and possibly improving overall recovery outcomes.

This study found that patients who had experienced only one stroke were less likely to be classified into the “trauma-sensitive profile” or the “pain-dominated profile.” The number of recurrent strokes has been identified as a critical determinant of disease threat perception (Chen et al., 2024); increased episodes substantially strengthen patients’ perceived risk of and concern about physical injury severity. This exaggerated perception of disease severity may manifest as catastrophic interpretations of exercise-induced physical sensations (Shimada et al., 2026). Patients with such perceptions may be more likely to no longer view exercise as a therapeutic means to facilitate recovery, but rather as a potential trigger for pain or discomfort. They may become hypervigilant to any bodily sensations during activity and tend to cease movement at the slightest sign of abnormality. Prolonged avoidance of exercise may be associated with cardiorespiratory deconditioning and exacerbate physical discomfort, which may perpetuate a vicious cycle. Therefore, healthcare professionals may consider prioritizing patients at risk of recurrence by strengthening early warning sign recognition and dynamically assessing exercise experiences to reinforce benefit perception, potentially supporting rehabilitation confidence and intrinsic motivation.

Higher symptom burden was associated with an increased likelihood of classification into the “pain-dominated profile,” consistent with Marques-Sule et al. (2025). Stroke patients report a mean of 12 symptoms within the first year, which is associated with impaired recovery and quality of life, and symptom burden has been identified as a factor associated with kinesiophobia (Huang et al., 2025). Co-occurring symptoms (e.g., pain, fatigue, and dizziness) may be catastrophically misinterpreted as indicators of re-injury, potentially reinforcing the belief that exercise will worsen their condition or trigger adverse events (Shimada et al., 2026). This may contribute to reduced or ceased activity, which in turn may be associated with functional decline and symptom aggravation, perpetuating a vicious cycle. Similar patterns have been reported in multiple sclerosis, where anxiety, low physical activity, and older age were independently associated with kinesiophobia (Mikuľáková et al., 2026); although MS and stroke differ, this supports the relevance of psychological and behavioral factors in neurological populations. Clinically, patients with extended disease courses or multisystem dysfunction may be prioritized. Prompt instruction on pathophysiology and rehabilitation, alongside individualized symptom control, may be worth considering to alleviate symptom-related distress and support patients’ belief in their recovery.

Additionally, higher rehabilitation confidence scores were associated with a lower likelihood of classification into either the “trauma-sensitive profile” or the “pain-dominated profile.” A previous study showed that rehabilitation confidence is a key factor associated with kinesiophobia (Özden et al., 2025). Specifically, patients with greater rehabilitation confidence were more likely to show stronger resilience, more active rehabilitation engagement, and superior functional outcomes (Bai and Chen, 2025). They tended to set feasible, gradual exercise targets and adapt their behavior when facing mild discomfort, rather than stopping altogether out of fear. Conversely, those with low confidence often held negative outcome expectations and felt incapable of managing exercise-related risks, such that even minor bodily sensations may be associated with helplessness and fear, which may contribute to avoidance. Thus, clinicians may consider highlighting the importance of progressive activity and promoting early, safe rehabilitation participation, with the aim of supporting patient confidence and recovery.

## 5 Limitations

First, convenience sampling from a single geographic region may introduce selection bias and limit generalizability to the broader Chinese stroke population. Second, the cross-sectional design permits correlation but not causation, calling for future longitudinal studies to clarify dynamic profile changes and causal pathways. Third, patients with severe skeletal musculoskeletal pathology or other disorders affecting exercise function were excluded, limiting generalizability to those with more pronounced motor impairment. Fourth, although functional independence was screened using the Barthel Index, other standardized measures of neurological and functional status were not systematically assessed; future studies should incorporate comprehensive assessments to disentangle these contributors. Fifth, the main variables were derived from self-reported measures, which are susceptible to recall bias. Sixth, LPA assigns individuals probabilistically; despite an entropy of 0.916, some misclassification cannot be excluded. The multinomial logistic regression model may have introduced sparse-cell or separation issues, although diagnostics did not indicate serious problems. Finally, the variable selection strategy based on univariate significance may have excluded clinically relevant confounders. Future research should include broader clinically relevant variables and objective assessments.

## 6 Conclusion

This study employed LPA to identify kinesiophobia profiles among middle-aged stroke patients during the recovery phase. The results revealed that kinesiophobia could be classified into three distinct profiles: the “self-regulation profile,” the “pain-dominated profile,” and the “trauma-sensitive profile.” Education level, family history of stroke, number of episodes, symptom burden, and rehabilitation confidence were significantly associated with kinesiophobia profiles. These findings suggest that healthcare providers can deliver more targeted interventions tailored to patients’ demographic characteristics and specific fear profiles. These findings also provide a foundation for future preventive strategies in exercise-based rehabilitation among this cohort.

## Data Availability

The raw data supporting the conclusions of this article will be made available by the authors, without undue reservation.
